# Mast cells are important regulator of acupoint sensitization via the secretion of tryptase, 5-hydroxytryptamine, and histamine

**DOI:** 10.1371/journal.pone.0194022

**Published:** 2018-03-07

**Authors:** Ning Ding, Jing Jiang, Pingping Qin, Qiaoxia Wang, Jiatong Hu, Zhigang Li

**Affiliations:** 1 School of Acupuncture, Moxibustion and Tuina, Beijing University of Chinese Medicine, Beijing, China; 2 School of Nursing, Beijing University of Chinese Medicine, Beijing, China; University of Pisa, ITALY

## Abstract

Mast cells (MCs) play a crucial role in mediating the establishment of networks among the circulatory, nervous and immune system at acupoints. However, the changes which occur in MCs during acupoint sensitization, i.e. the dynamic transformation of an acupoint from a "silenced" to an "activated" status, remain uncharacterized. To investigate the morphological and functional changes of MCs as an aid to understanding the cellular mechanism underlying acupoint sensitization, a rat model of knee osteoarthritis (OA) was induced by an injection of mono-iodoacetate (MIA) on day 0. On day 14, toluidine blue and immunofluorescence staining were used to observe the recruitment and degranulation of MCs and the release of mast cell co-expressed mediators: tryptase, 5-hydroxytryptamine (5-HT) and histamine (HA) at the acupoints Yanglingquan (GB34), Heding (EX-LE2) and Weizhong (BL40). Results showed that the number of MCs as well as the percentages of degranulated and extensively degranulated MCs at the acupoints GB34 and EX-LE2 in the light (A), mild (B), heavy (C) osteoarthritis groups were larger than those in the normal control (N) and normal saline (NS) groups (p < 0.01). Comparisons among the A, B and C groups suggested that the number and the degranulation extent of the MCs at the acupoints GB34 and EX-LE2 were positively correlated with the severity of the disease. Some MCs in the A, B and C group showed the release of 5-HT, HA, and tryptase in degranulation at the acupoints GB34 and EX-LE2. Such changes in MCs were not observed at the acupoint BL40. In conclusion, this study confirmed that acupoint sensitization is associated with the increase in recruitment and degranulation levels of MCs on a acupoint-specific and disease severity-dependent manner. The release of tryptase, 5-HT, and HA during MC degranulation is likely to be one of the cellular mechanisms occurring during acupoint sensitization.

## Introduction

Mast cells (MCs) are important components of the immune system, widely viewed as potential effector cells in acupuncture. Previous studies showed that the frequency and the type of function of MCs in the colon mediate the effects accomplished by acupuncture on the improvement of the irritable bowel syndrome [1–2]. Additionally, the link between the recruitment and degranulation of MCs at acupoints have been observed histologically as a response to acupuncture therapy [3–5].

It is worthwhile pointing out that the location and functional status of acupoints are considered dynamic within a certain range of time and space, varying with different physiological and pathological conditions [6–7]. It has also been reported that MCs not only induce a neuro-immuno response by the release of inflammatory mediators following acupuncture; they also play a crucial role in the cross-talk among the circulatory, nervous and immune networks at acupoints [8–9]. However, the current studies were limited to the post-acupuncture intervention period and focused on the response of MCs’ to acupuncture at acupoints or in effector organs. The functional and morphological changes of MCs during the acupoint sensitization upon different diseases and levels of disease severity have been neglected to a certain extent and remain largely elusive. The dynamics of the acupoint response to disease are confined to the term “acupoint sensitization”. It refers to the dynamic transformation of an acupoint from a "silenced status" (as induced by healthy conditions) to an "activated status” (as induced by disease) [7,10]. Previous studies showed that acupoint sensitization markedly affects the size of an acupoint’s receptive field as well as the sensitivity and response-capacity of an acupoint to the therapeutic effects of acupuncture [11–12]. Therefore, acupoint sensitization is an important prerequisite that enables it to perceive and respond to outside stimuli, allowing it to contribute to the restoring of body functions weakened by disease. In addition, as disease indicators located on the body surface, sensitized acupoints can be used in clinical prognosis and disease monitoring practices [13–14].

Therefore, the characterization of the morphological and functional changes of MCs during acupoint sensitization, which occur as a response to disease at acupoints, will help to elucidate the mechanism which leads to the reconfiguration of the circulatory, nervous and immune networks at acupoints during sensitization. Ultimately, this study aims to contribute to the understanding of the scientific principles underlying acupuncture and widen the use of acupuncture practices in clinical diagnosis, disease monitoring and treatment.

In this study, a rat model of knee osteoarthritis (OA) was induced by mono-iodoacetate (MIA). Safranine-O and Fast Green staining were used to assess the level of damage caused by MIA-induced osteoarthritis. Subsequently, morphological and functional changes of MCs were assessed during acupoint sensitization using toluidine blue and immunofluorescence staining. Our data can be added to the debate on the role MCs as mediators of acupoint sensitization and supports the use of acupoints in clinical practices as disease indicators and as effector areas for acupuncture.

## Materials and methods

### Experimental animals

Sprague-dawley (SD) rats were purchased from the SPF (Beijing) Biotechnology Co., Ltd (Animal Lot: SCXK(Jing)2016-0002). SD rats weighed 180.0±10.0 g. The animals were housed in a fenced facility in the Experimental Animal Center of Beijing University of Traditional Chinese Medicine at a controlled temperature (24±2°C) and under a 12-h dark/light cycle, with sterile drinking water and a standard pellet diet available ad libitum. All rats were acclimatized to the environment for 7 days prior to experimentation, and all experimental procedures complied with the guidelines of the “Principles of Laboratory Animal Care” formulated by the National Institute of Health and the legislation of the People’s Republic of China for the use and care of laboratory animals. The experimental protocols were approved by the Beijing university of Chinese Medicine Medicine and Animal Ethics Committee (S2 Text). Efforts were made to minimize the number of animal uses and the suffering of the experimental animals. The laboratory protocols can be found at http://dx.doi.org/10.17504/protocols.io.mmpc45n.

### Animal groups and interventions

Forty SD male rats were divided into five groups randomly (n = 8 per group): the normal control (N) group, the normal saline group (NS), the light osteoarthritis group (A), the mild osteoarthritis group (B) and the heavy osteoarthritis group (C).

According to Guingamp [15], MIA (Sigma, St. Louis, USA) was dissolved in 50μl of sterile saline water. MIA solution was injected into the right knee joint through the infrapatellar ligament. On day 0, the rats in the A, B and C group were injected with 0.3, 1, 3 mg MIA respectively, and rats in the NS group were injected with 50 μl of saline solution, in accordance with Pomonis[16]. No intervention was carried out in the N group.

### Safranine-O and fast green staining

At day 14, 6 rats in each group were killed by an intraperitoneal injection of pentobarbital (150 mg/kg body weight). The knee joints were extracted and fixed in 10% neutral formalin. Then, the knee joints were placed in 5% formic acid for 10 days for demineralization, dehydrated, embedded in paraffin and sectioned with a 5-μm slicer. Subsequently, the sections were dewaxed, dehydrated, and stained in 1% fast green for 1.5 min and then differentiated by acetic acid. Next, the sections were stained by 0.5% safranine-O for 1.5 min, differentiated by ethanol, and washed with tap-water. After vitrification with dimethylbenzene and neutral balata fixation, the sections were observed under a light microscope (CX31, Olympus Corporation, Japan), and subjected to a quantitative osteoarthritis cartilage histological analysis based on the method described by Pritzker [17].

### Toluidine blue staining

At day 14, the skin and subcutis at the acupoints Yanglingquan (GB34), Heding (EX-LE2) and Weizhong (BL40) were dissected from 6 rats killed by an intraperitoneal injection of pentobarbital before the knee joints were taken. The location of the acupoints GB34, EX-LE2, BL40 was described in S1 Text. The dimensions of each tissue fragment were 1.5 × 1.5 × 1.5 mm. The tissues were fixed in 10% neutral formalin, dehydrated, embedded in paraffin and sectioned with a 4-μm slicer. Subsequently, the sections were dewaxed, dehydrated and stained in 0.5% toluidine blue for 30 min and then washed with tap-water. After vitrification with dimethylbenzene and neutral balata fixation, the sections were observed under a light microscope (CX31, Olympus Corporation, Japan), at a 40 × magnification (field diameter 0.5 mm). The number of MCs (per 0.2 mm^2^) and their degree of degranulation at the subcutaneous connective tissue were recorded. In each acupoint, six different fields obtained from six separate samples were examined. MCs were classified as "extensively degranulated" (>50% of the cytoplasmic granules exhibiting fusion, staining alterations, and extrusion from the cell), "moderately degranulated" (10–50% of the granules exhibiting fusion or discharge), or "normal" as described by Martin [18]. The percentage of degranulated MCs was the number of degranulated MCs divided by the total number of MCs, the percentage of extensively degranulated MCs was the number of extensively degranulated MCs divided by the total number of MCs.

### Immunofluorescence staining

At day 14, 2 rats in each group were anesthetized by an intraperitoneal injection of pentobarbital (40 mg/kg body weight) and perfused with 4% paraformaldehyde. The skin and subcutis at GB34, EX-LE2, and BL40 were dissected and tissue fragment of 1.5 × 1.5 × 1.5 mm were prepared. The tissues were fixed in 4% paraformaldehyde for 2.5 h, and dehydrated by 25% sucrose for 2 days. Frozen 10-μm sections were sliced by a freezing microtome (Leica Corporation, Germany) at -25°C. The primary antibodies included the mouse monoclonal mast cell tryptase antibody (1:100, Abcam, USA), the goat polyclonal 5-hydroxytryptamine (5-HT) antibody (1:100, Abcam, USA), and the rabbit polyclonal histamine (HA) antibody (1:200, LSBio, USA). Donkey Anti-Mouse IgG Alexa Fluor 488 (1:200, Abcam, USA), Donkey Anti-Goat IgG Alexa Fluor 594 (1:200, Abcam, USA), and Donkey Anti-rabbit IgG Alexa Fluor 594 (1:200, Abcam, USA) were used as corresponding secondary antibodies. The solution 4’,6-diamidino-2-phenylindole dihydrochloride (DAPI, Solarbio, Beijing, China) was applied for counterstaining. For double immunohistochemical staining, the sections were washed in 0.1 M PB (pH = 7.4) and blocked in 0.1 M PB (pH = 7.4) containing 3% normal donkey serum and 0.5% Triton X-100 for 30 min. Next, the sections were treated with mouse monoclonal mast cell tryptase antibody and goat polyclonal 5-HT antibody and incubated overnight at 4°C. After PB washes, the sections were exposed to Donkey Anti-Mouse IgG Alexa Fluor 488 and Donkey Anti-Goat IgG Alexa Fluor 594 for 2 hours. Subsequently, the sections were washed with PB and stained with DAPI for 6 minutes. After washing with PB, the sections were observed under a confocal laser scanning microscope (FV1000, Olympus Corporation, Japan). The number of MCs which were double stained and their degree of degranulation were recorded at a 60 × magnification. In each acupoint, two different fields obtained from two separate samples were examined. Double immunohistochemical staining using the same staining protocol was undertaken, to examine the co-expression of mast cell tryptase and HA.

### Statistical analysis

The statistical analysis was performed using the SPSS software, version 17.0 (SPSS, Inc., Chicago, IL, USA), and the data were expressed as the mean ± standard deviation. A one-way ANOVA was used after the normal distribution and homogeneity of variance were confirmed. For the non-normally distributed data or for data with heterogeneous variance, a kruskal-wallis ANOVA was used. The LSD method was applied for pairwise comparisons. Statistical significance was set to p< 0.05 and high statistical significance was set to p< 0.01.

## Results

### Safranine-O and fast green staining of articular cartilage and OA scores

**Fig 1:**
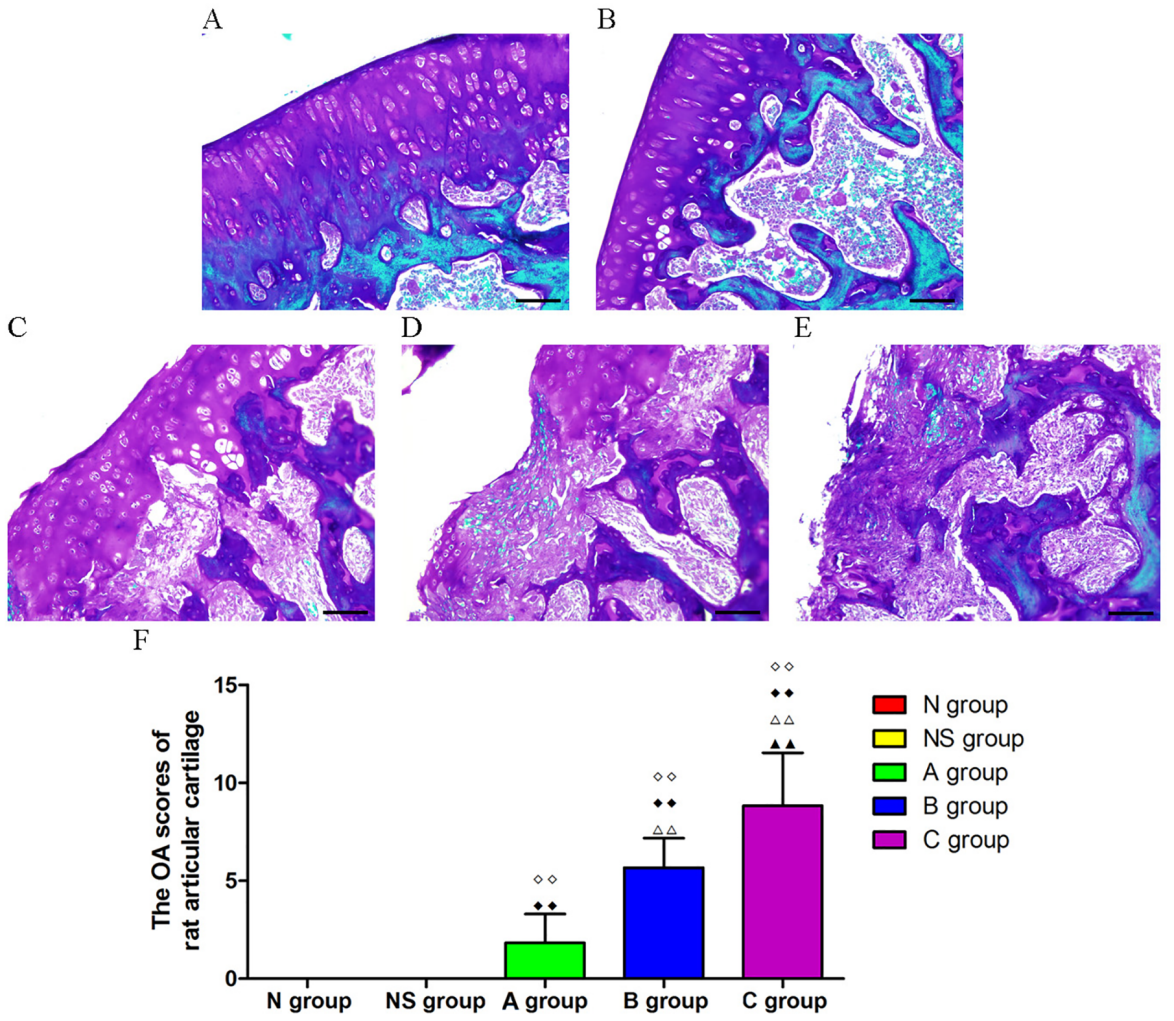
Assessment of suitability of the MIA-induced knee osteoarthritis rat model. (A-E) Representative images from the safranine-O and fast green staining of the N group, the NS group, the A group, the B group and the C group. Scale bar is 100 μm. (F) Comparison of the OA scores as assessed on articular cartilage of model rats in each group. Data were showed as means ± SD, n = 6, ◇◇P < 0.01 vs the N group, ◆◆P < 0.01 vs the NS group, ΔΔP < 0.01 vs the A group, ^▲▲^P < 0.01 vs the B group.

The results of the safranine-O and Fast Green staining and associated OA quantitative measurements (OA scores) of the articular cartilage sections of model rats are presented in Fig 1. The articular surface and cartilage morphology of the N and NS groups were intact and no damage was observed. Surface discontinuity, superficial fibrillation, cationic stain matrix depletion of the upper 1/3 of the cartilage, as well as hypertrophy of cartilage cells were observed in the A group, where the cartilage involvement was lower than 10%. The cartilage pathological changes in the B group included erosion and matrix loss, delamination of the superficial layer, and mid-layer cyst formation. The range of damage varied from 10–25%, sometimes reaching 50%. Denudation and deformation of the cartilage, as well as the presence of reparative tissue including fibrocartilage within the denuded surface, could be observed in the C group. The cartilage involvement was 25–50% and greater. OA scores in the A, B, and C groups were significantly higher than those in the N and NS groups (p < 0.01). As expected the OA scores increased with the use of higher concentrations of MIA. Accordingly, the OA score of the B group was higher than that of the A group (p < 0.01). The OA score of the C group was higher than those of the A and B groups (p < 0.01).

### Changes in number and degranulation levels of MCs at the acupoints GB34, EX-LE2, and BL40 during sensitization

**Fig 2:**
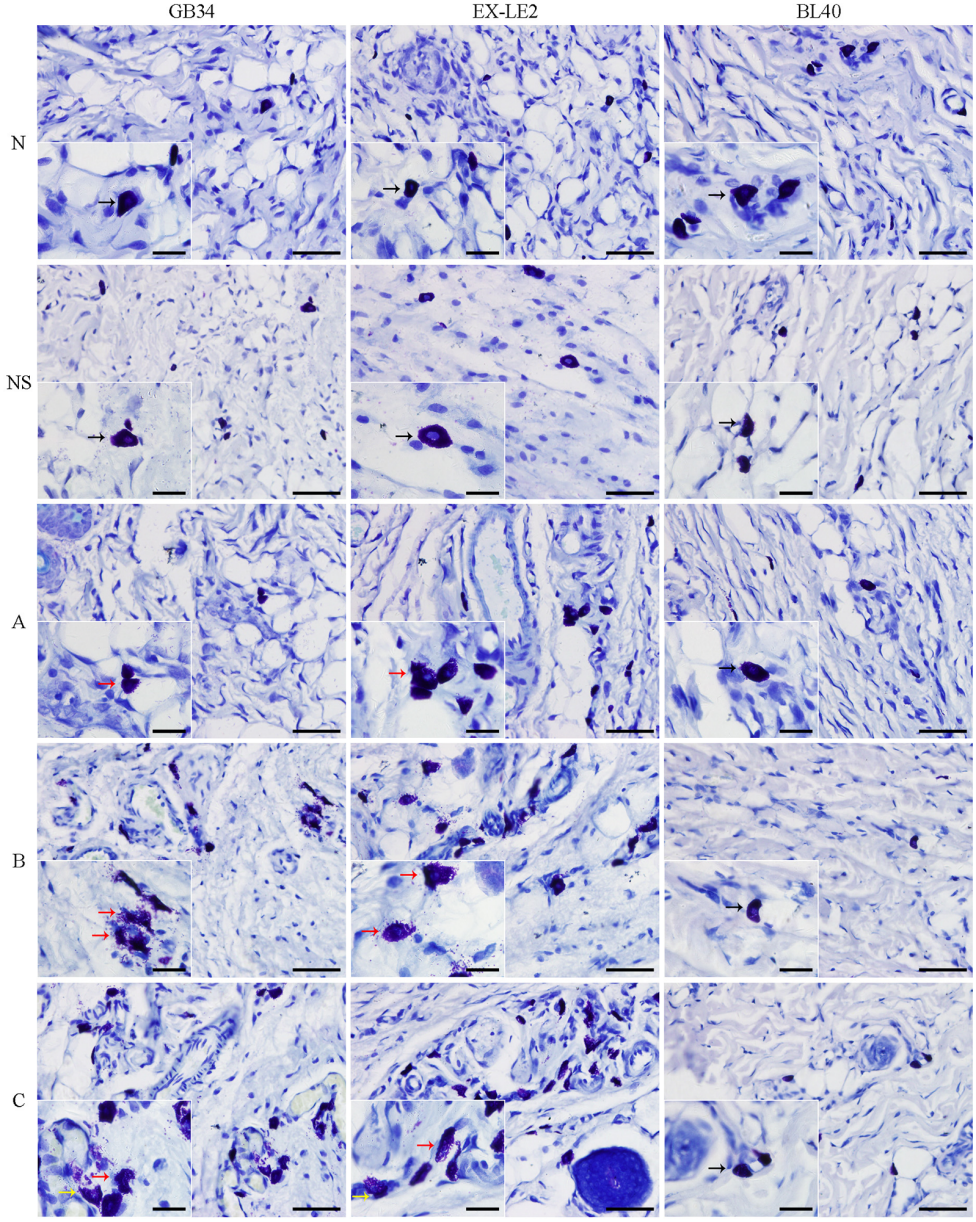
Representative images of toluidine blue staining of each rat group. The MCs were exhibited round or elliptic morphologies, their nuclei were round and the cytoplasm was homogeneously spotted with basophilic purple granules. Some MCs at the acupoints GB34 and EX-LE2 in the A, B and C group showed a large number of bluish violet granules distributed in between the cells and arranged in bundles or bands (degranulation). The normal, moderately degranulated and extensively degranulated MCs are shown by black, red and yellow arrows. Scale bar is 40, 20 μm respectively.

Toluidine blue staining of the MCs at the acupoints GB34, EX-LE2 and BL40 are presented in Fig 2. The MCs were scattered in the subcutaneous connective tissue. The number of MCs at the acupoints GB34, EX-LE2, and BL40 in the N and NS groups was small. On the contrary, a higher number of MCs with degranulation was observed at the acupoints GB34 and EX-LE2 in the A, B and C groups. The number of MCs and the extent of degranulation of MCs increased with the severity of disease in both the GB34 and EX-LE2 acupoints. The changes in MCs at the acupoint BL40 were not observed.

**Fig 3:**
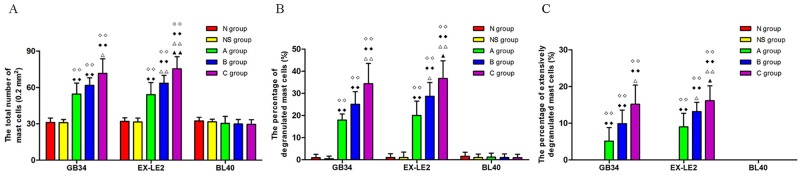
The number of MCs and degranulation levels in each rat group. (A) The number of MCs in all groups. (B) The percentages of degranulated MCs in all groups. (C) The percentage of extensively degranulated MCs in all groups. Data were showed as means ± SD, n = 6, ◇◇P < 0.01 vs the N group, ◆◆P < 0.01 vs the NS group, ΔΔP < 0.01, ΔP < 0.05 vs the A group, ^▲▲^P < 0.01, ^▲^P < 0.05 vs the B group.

The quantitative representation of MCs changes as a response to knee osteoarthritis is shown in Fig 3. The number of MCs, as well as the percentages of degranulated and extensively degranulated MCs, at the GB34 and EX-LE2 acupoints in the A, B, and C groups were significantly higher than those in the N and NS groups (p < 0.01). Comparisons between levels of disease (differences among the A, B and C groups) showed that the number and the degranulation extent of the MCs correlated with the severity of the disease. In particular, the number of MCs, as well as the percentages of degranulated MCs and extensively degranulated MCs, at EX-LE2 in the A group were lower than those in the B group, which in turn were lower than those in the C Group (p < 0.01 or p < 0.05). The number of MCs, as well as the percentages of degranulated MCs and extensively degranulated MCs, at GB34 in the A group were lower than those in the C group (p < 0.01 or p < 0.05). No quantitative differences in MCs were observed at BL40 as a response to knee osteoarthritis.

### Changes in MCs co-expression levels of tryptase, 5-HT, and HA at the GB34, EX-LE2, and BL40 acupoints during sensitization

**Fig 4:**
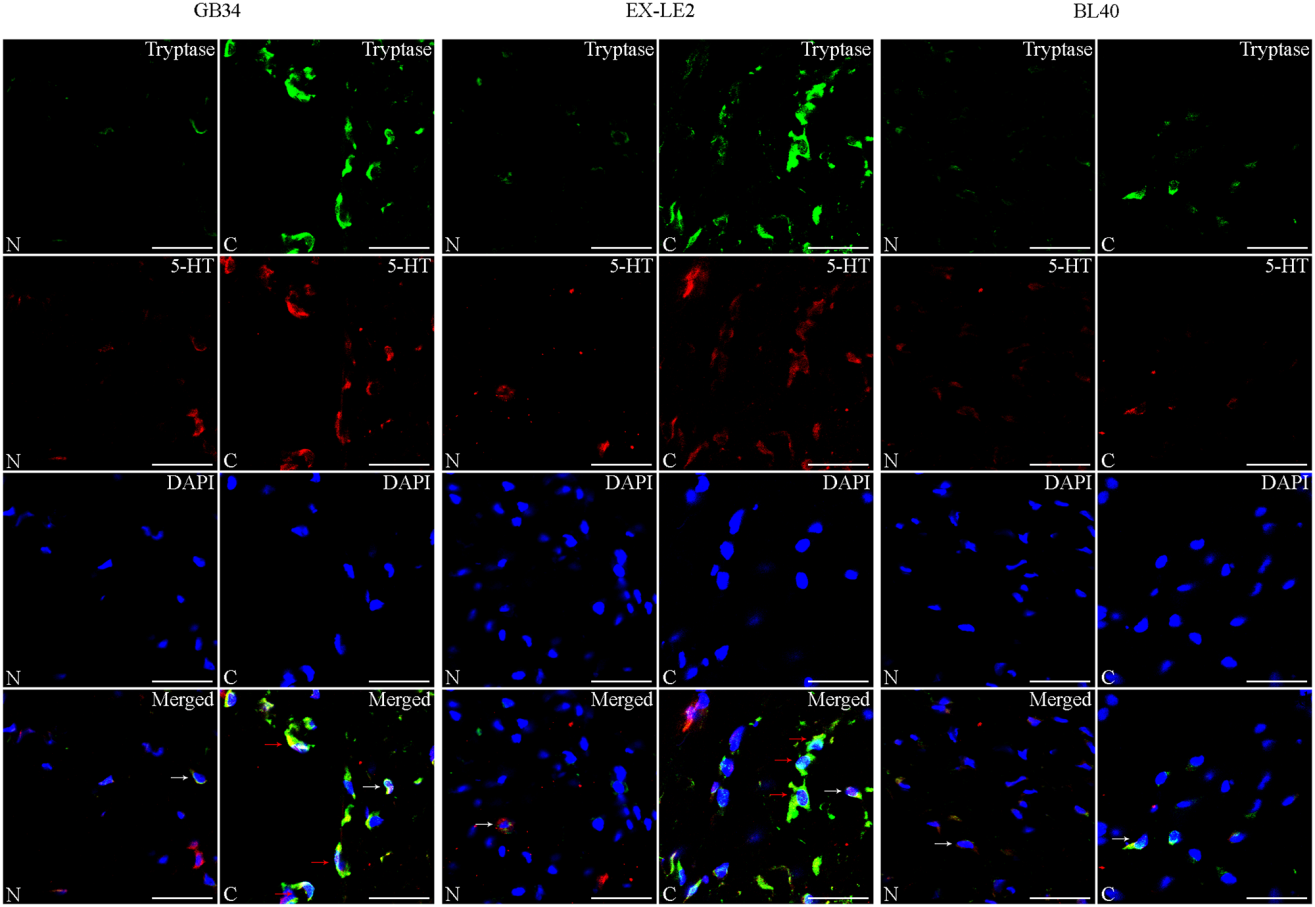
Representative images of immunofluorescence staining of mast cell tryptase (green) and co-expressed 5-HT (red) in the N and C group. The MCs-produced tryptase, mainly expressed in the cytoplasm, was green fluorescence labeled and the co-expression of 5-HT was red fluorescence labeled. The positively stained cells, degranulated MCs are shown by white, red arrows. Scale bar is 40 μm.

**Fig 5:**
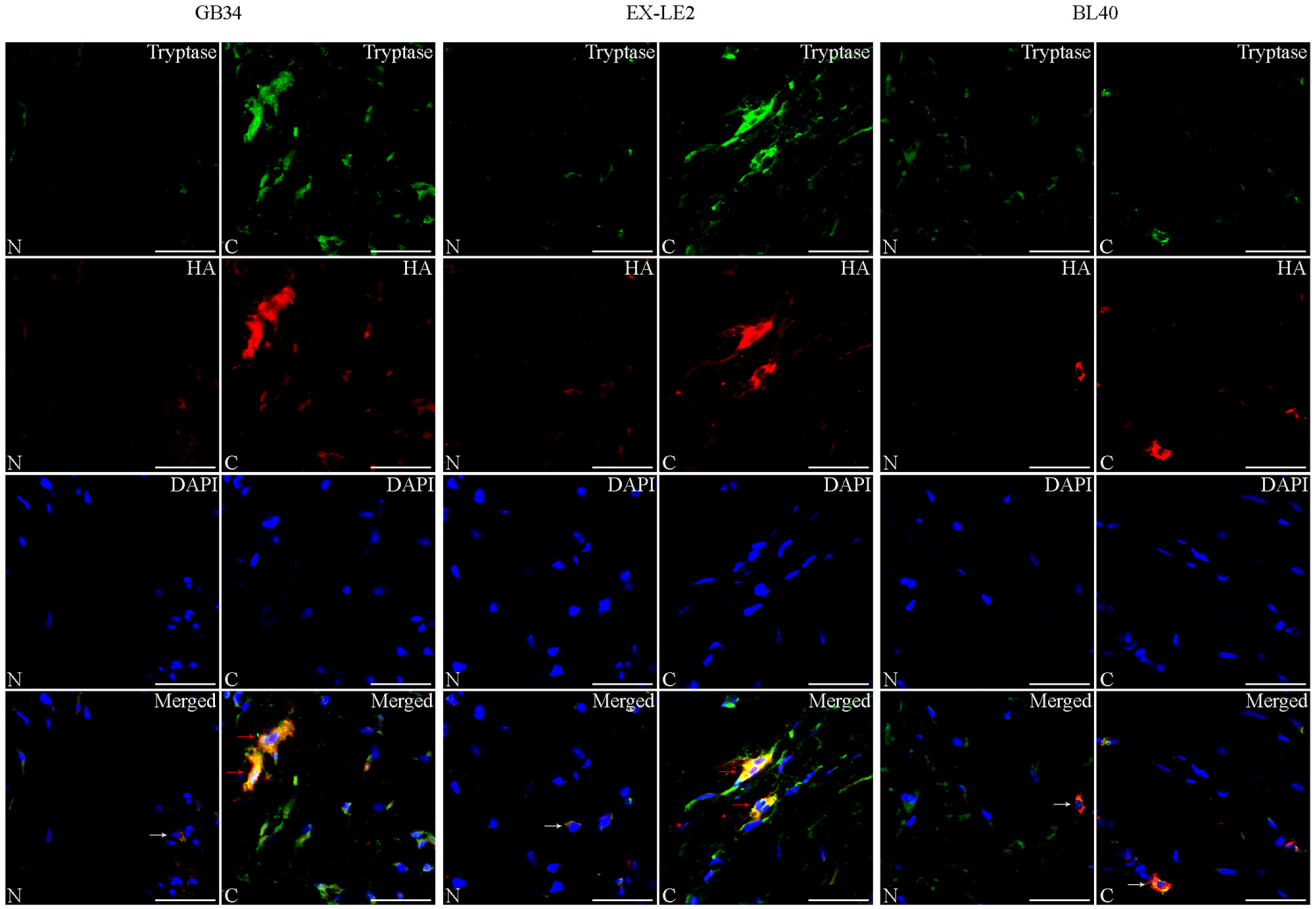
Representative immunofluorescence staining of mast cell tryptase (green), and co-expressed HA (red) in the N and C group. The MCs-produced tryptase, mainly expressed in the cytoplasm, was green fluorescence labeled and the co-expression of HA was red fluorescence labeled. The positively stained cells, degranulated MCs are shown by white, red arrows. Scale bar is 40 μm.

Immunofluorescence staining of tryptase, 5-HT, and HA are presented in Figs 4 and 5, S1 and S2 Figs. There were fewer positively stained tryptase MCs in the N and NS groups. In these groups, the co-expressed level of 5-HT, and HA was low. The degranulation of MCs were absent, which is in accordance with the results from toluidine blue staining. However, in the A, B and C groups, there was an increase in positively stained cells both in number and as in the intensity of staining (representing higher levels of expression of tryptase, 5-HT, and HA) at the acupoints GB34 and EX-LE2. In the A, B and C groups, some MCs at the acupoints GB34 and EX-LE2 showed the release of 5-HT, HA and tryptase into the extracellular region, which is typical of MCs degranulation. In addition, the number of immunofluorescence stained MCs, as well as their degranulation levels at the acupoints GB34 and EX-LE2 in the A, B and C groups, tended to that increase with the degree of disease severity. No differences in MCs immunofluorescence staining were observed at the acupoint BL40 as a response to knee osteoarthritis.

## Discussion

Acupuncture has been widely used all over the world as an effective physical therapy for knee osteoarthritis. A growing body of evidence has demonstrated that acupuncture can alleviate pain in knee osteoarthritis and improve knee function [19–20] with few adverse effects [21–22]. In terms of acupoints selection for knee osteoarthritis acupuncture-based treatment, the acupoints around the knee are preferred. Specifically, for the treatment of knee osteoarthritis the acupoints GB34 [23–24], EX-LE2 [25] and BL40 [26] are commonly selected. According to Traditional Chinese Medicine, acupoints have the dual function of reflecting disease severity and of restoring the function of the disease-affected body parts [10]. The acupoints analyzed in this study, closely located around the knee, were selected in order to elucidate on the functional and morphological changes of acupoint during its sensitization to knee osteoarthritis and to add additional data to the debate concerning their use as disease indicators, in clinical prognosis and disease monitoring practices.

Our results obtained using the safranine-O and fast green staining showed that the articular cartilages in the A, B, and C groups suffered significantly more damage than those in the N and the NS groups, as specified by their OA scores. These results indicate that different osteoarthritis levels (light, mild and heavy) were successfully induced by injecting different doses of MIA to rats. Therefore, we created a reliable OA rat model for follow up experiments.

The results of the toluidine blue staining analysis showed that the number of MCs and the extent of MCs degranulation at the acupoints GB34 and EX-LE2 in the A, B, and C groups were drastically higher than those in the N and NS groups. These results indicate that the acupoint sensitization is associated with an increase in MCs recruitment and degranulation levels. The fact that the increase in the recruitment and degranulation level of MCs were not observed at BL40, demonstrates that the morphological and functional changes of MCs during acupoint sensitization are acupoint-specific. In addition, the number and degranulation of the MCs increased with the degree in disease severity, primarily at EX-LE2, but closely followed by GB34, suggesting that the degree of acupoint sensitization is different among the sensitizated acupoints.

The differential increased in the levels of acupoint sensitization of GB34 and EX-LE2, as opposed to that of BL40, are in agreement with the current acupoint selection for knee osteoarthritis in acupuncture [27–28]. In addition, and as mentioned before, the function of acupoints involves being both a reflector of disease severity and a physical point to treating disorders. Thus, the acupoints which are least frequently used in clinical practice correspond to acupoints with a lower sensitization response to disease. Our results on the acupoint BL40 conform to the existing empirical knowledge that BL40 is less implicated in in the response to knee osteoarthritis (and is therefore less used) than GB34 and EX-LE2 in current acupuncture practices. Our results as well as the acupuncture empirical knowledge suggest a relatively lower specificity of BL40 to knee osteoarthritis. On the other hand, this study strongly suggests that early diagnosis as well as disease progression monitoring can be achieved through the observation of the dynamical changes occurring during acupoint sensitization at particular acupoints since the degrees of acupoint sensitization and disease severity were closely correlated. Also, this study suggests that careful acupoint selection and monitoring might improve the clinical effect of acupuncture treatments.

Regarding the possible mechanisms underlying an increase in the MCs recruitment and degranulation levels during acupoint sensitization, some studies have focused on the structure of the MCs, nerve fibers and blood vessels in the acupoint in order to understand possible interactions between the circulatory, nervous and immune system from a histological perspective [29–30]. In particular, adhesion molecules [31], angiogenic factors [32], lipid mediators [33], which upon expression in or release from the endothelial cells, have been reported capable of promoting recruitment of MCs. Neuropeptides released by nerve endings have also been reported to activate MCs [34–35]. In view of the previous findings and the current results, we propose that the increase in recruitment and degranulation levels of MCs during acupoint sensitization may be the result of complex interactions between the circulatory, nervous and immune systems, which are location-, and disease severity-dependent. However, the unraveling of the cross-talk among the different systems during acupoint sensitization requires further investigation.

The release of tryptase from the mast cell secretory granules is typical of MC degranulation [36]. Our results of the immunofluorescence staining analysis further confirmed that MC degranulation occurred at acupoints GB34 and EX-LE2 affected by knee osteoarthritis (but not at BL40). Furthermore, the number and degranulation of the MCs increased with the severity of disease as indicated by the toluidine blue staining at the acupoints GB34 and EX-LE2. Importantly, this study confirmed, for the first time, the positively expression of 5-HT and HA in MCs degranulation during acupoint sensitization. Therefore, in addition to the increase in expression of tryptase, the increase in 5-HT and HA also occurs during acupoint sensitization. As neurotransmitters and immune mediators, 5-HT and HA are important regulators of the microcirculatory system [37–38] and involved in allergy [39–40]. These factors, 5-HT and HA, have been attributed a potential role in the exchange of information between MCs and nerve terminals [41]. As a major constituent of the mast cell secretory granules, mast cell-released tryptase has the physiological role of promoting angiogenesis and participating in local inflammatory reactions [36, 42]. These findings seem to indicate that during acupoint sensitization, MCs have a role in regulating the interactions between the circulatory, nervous and immune system via the release of tryptase, 5-HT, and HA during degranulation of MCs located at specific acupoints, creating a cross-talk between the blood-vessels, nerves and immune factors at these locations.

Previous studies showed the implication of 5-HT, HA and their related receptors in the development of skin diseases [43–44], and tryptase is related to the itching symptom of chronic dermatitis [45]. Additionally, 5-HT, HA and tryptase are involved in peripheral mechanisms of pain and can modulate pain transmission by several mechanisms [46–48]. All bioeffects associated with the release of 5-HT, HA, and tryptase from MCs at specific locations help explain the acute tenderness and dermatosis of acupoints. Additionally, meridians have been regarded as lower-resistance passages for the chemotactic migration of cells, whereas MCs can indeed migrate longitudinally along the meridians [49]. Moreover, the release of 5-HT, HA, and tryptase from MCs have been reported to induce the migration and activation of MCs [50–52]. In summary, we propose that the release of 5-HT, HA, and tryptase during degranulation of MCs might directly affect the circulatory, nervous and immune networks at the diseased affected acupoints primarily but that their effect might extend throughout a meridian, thus being the physiological mechanism underlying acupoint sensitization, in a multilevel response to disease.

## Conclusions

The results obtained with our rat model of knee osteoarthritis indicate that MCs are involved in acupoint sensitization. An increase in recruitment and degranulation levels of MCs during acupoint sensitization was observed. Moreover, our results strongly suggest that the increase in recruitment and degranulation levels of MCs were positively correlated with the severity of disease on a location-dependent manner. The fact that acupoint sensitization was correlated with disease severity and was location-specific might give acupoints a role in clinical practice as disease indicators, specifically in diagnosis and monitoring. Furthermore, this study was able to confirm, for the first time, the positively expression of 5-HT and HA in MCs degranulation during acupoint sensitization. The release of 5-HT, HA, and tryptase during degranulation of MCs is likely to underlie the cross-talk between the circulatory, nervous and immune systems at acupoints. Therefore, MCs might play a trigger role in acupoint sensitization.

## Supporting information

S1 Fig Representative images of immunofluorescence staining of mast cell tryptase (green) and co-expressed 5-HT (red) in the NS, A and B group. (PDF)

S2 Fig Representative images of immunofluorescence staining of mast cell tryptase (green) and co-expressed HA (red) in the NS, A and B group. (PDF)

S1 Text Location of the acupoints GB34, EX-LE2, BL40. (PDF)

S2 Text Ethics committee approval files. (PDF)
