# Supplementary figures and images for: Mast cells are important regulator of acupoint sensitization via the secretion of tryptase, 5-hydroxytryptamine, and histamine
Source: PLoS One. 2018 Mar 7;13(3):e0194022. doi: 10.1371/journal.pone.0194022 (PMC5841809; doi:10.1371/journal.pone.0194022)

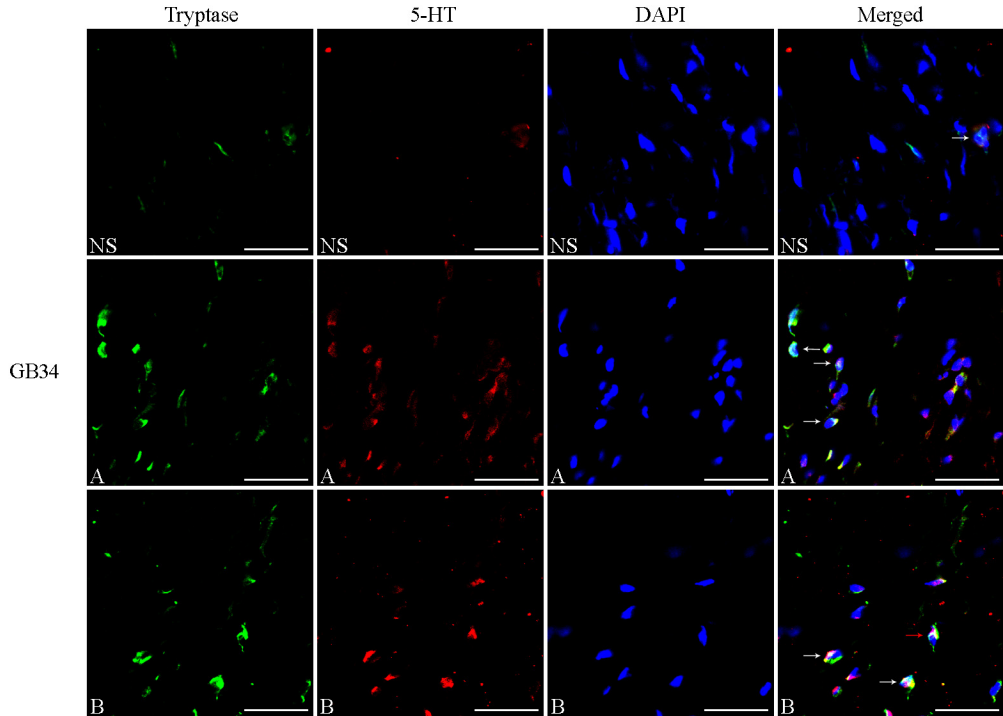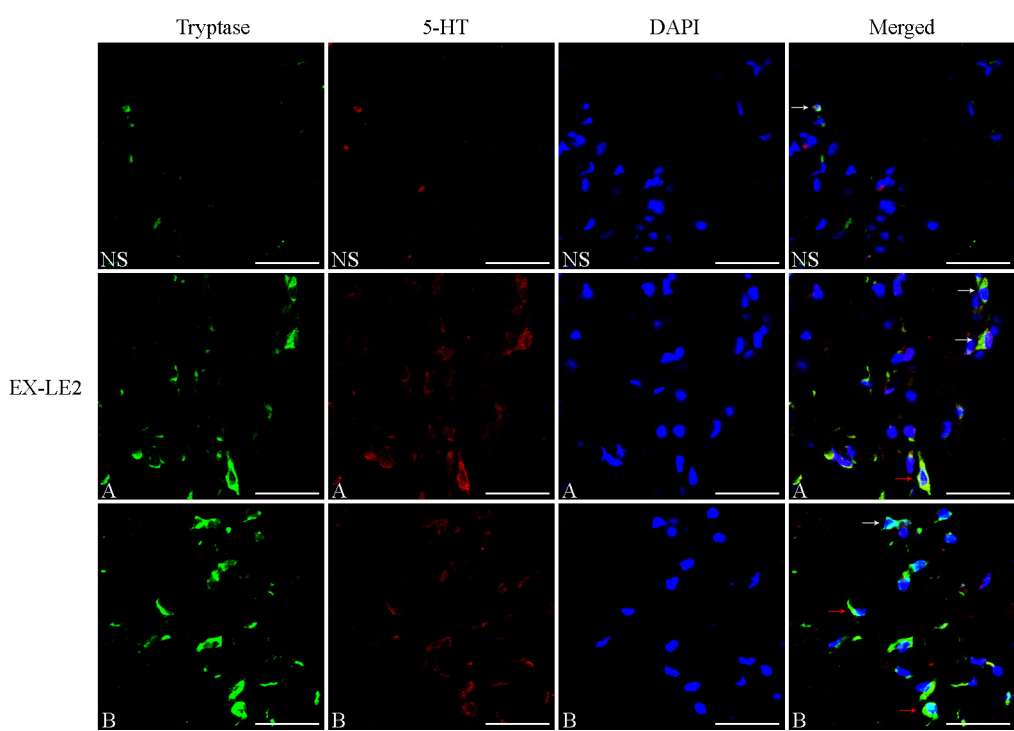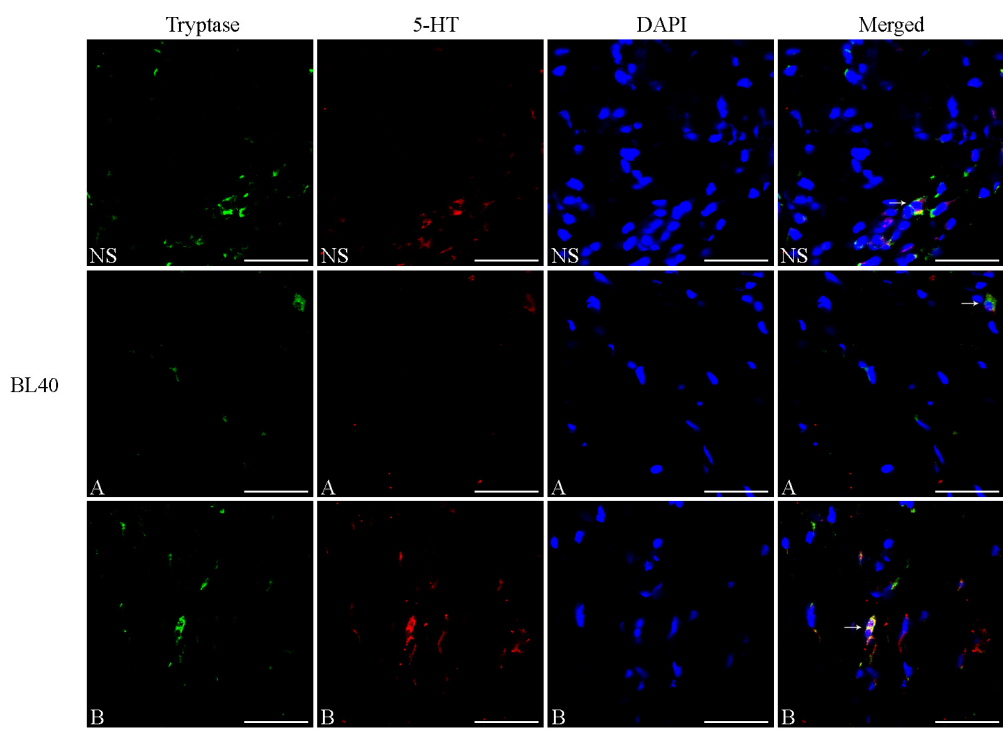

Supplement: S1 Fig — (PDF) [file pone.0194022.s001.pdf]

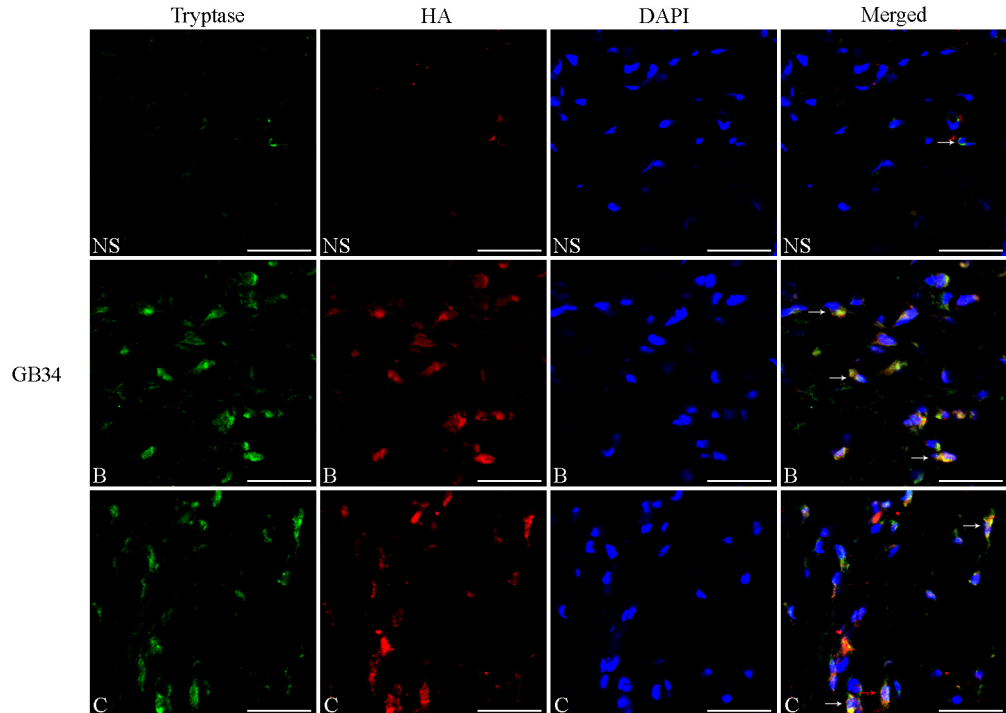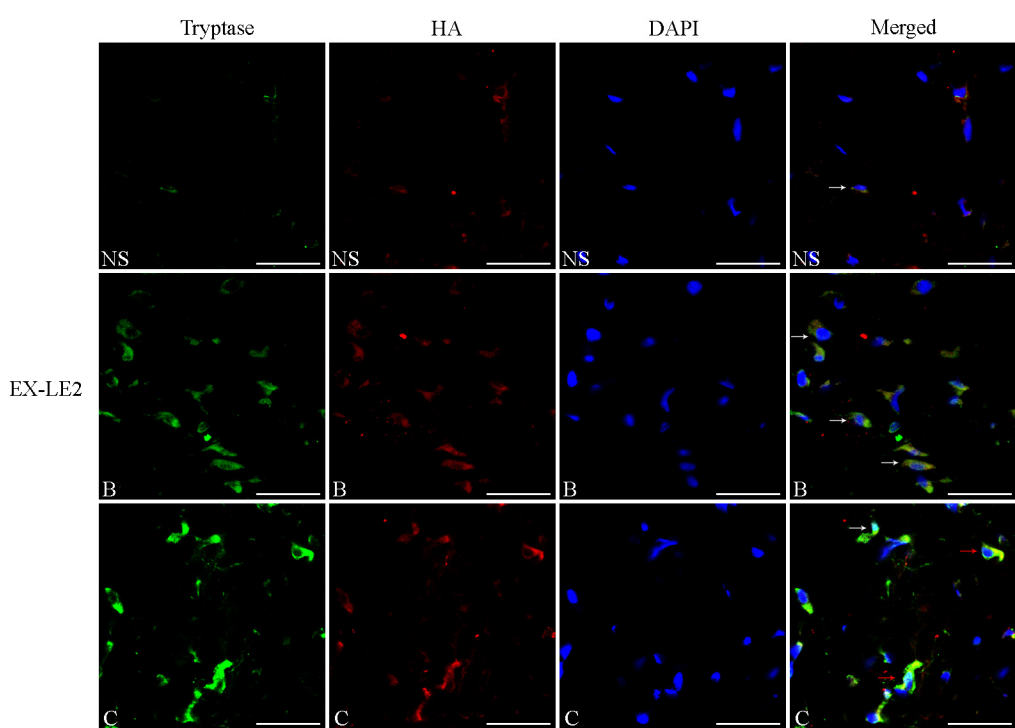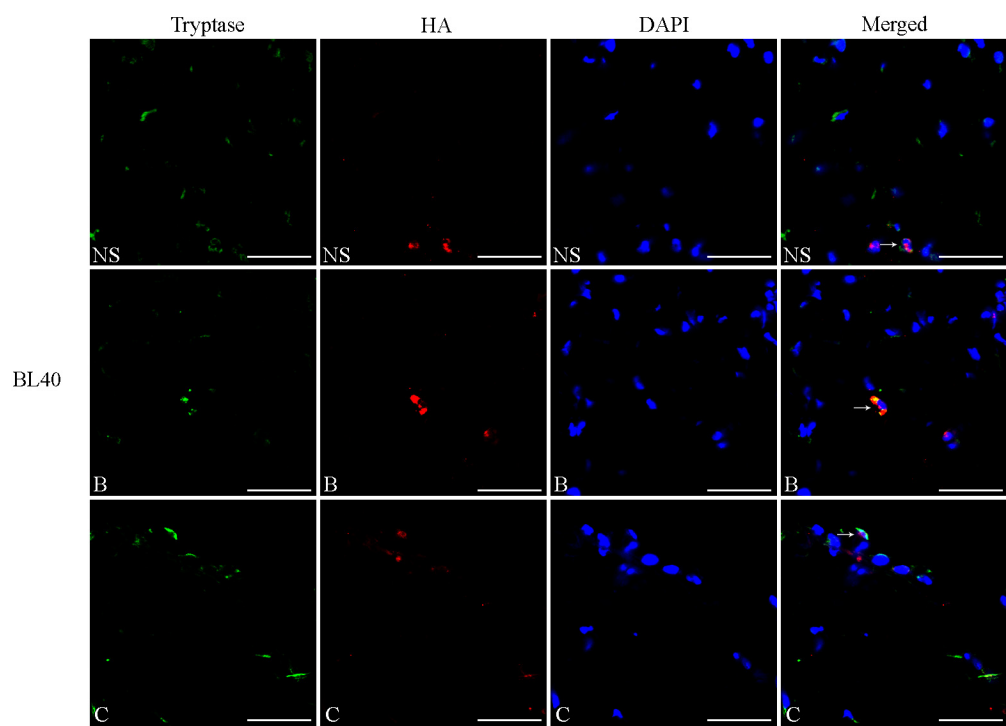

Supplement: S2 Fig — (PDF) [file pone.0194022.s002.pdf]
