# Supplementary material for: Mast cells are important regulator of acupoint sensitization via the secretion of tryptase, 5-hydroxytryptamine, and histamine
Source: PLoS One. 2018 Mar 7;13(3):e0194022. doi: 10.1371/journal.pone.0194022 (PMC5841809; doi:10.1371/journal.pone.0194022)
Supplement: S1 Text — (PDF) [file pone.0194022.s003.pdf]

Yanglingquan (GB34) is located in the depression below the capitulum fibulae posterolateral to the knee joint.

Heding (EX-LE2) is located in the depression at the midpoint of the superior border of the patella.

Weizhong (BL40) is located at the back of the knee, on the popliteal crease, in a depression midway between the tendons of biceps femoris and semitendinosus.
