# Supplementary material for: Mast cells are important regulator of acupoint sensitization via the secretion of tryptase, 5-hydroxytryptamine, and histamine
Source: PLoS One. 2018 Mar 7;13(3):e0194022. doi: 10.1371/journal.pone.0194022 (PMC5841809; doi:10.1371/journal.pone.0194022)
Supplement: S2 Text — (PDF) [file pone.0194022.s004.pdf]

# 北京中医药大学动物实验伦理审查表

编号(No)：BUCM- 4-2017051717-2017

<table>
<tr><td rowspan="8">申请人填写的相关信息</td><td colspan="2">申请人：丁宁</td><td colspan="2">实验动物上岗证号：1115010900264</td></tr>
<tr><td colspan="4">实验名称：穴位敏化的成像研究</td></tr>
<tr><td rowspan="3">动物情况</td><td colspan="3">动物来源：斯贝福</td></tr>
<tr><td colspan="2">品种品系：SD 大鼠</td><td>等级：清洁级</td></tr>
<tr><td colspan="2">数量100只(♀ 0 只；♂100只)</td><td>申请日期：2017年5月15日</td></tr>
<tr><td colspan="4">实验要点：（包括疾病模型造模、手术方法、实验结束后处死动物的方法等）<br>1 造模、手术方法：<br>采用单碘乙酸盐（MIA）法制备大鼠膝骨关节炎模型，具体方法为：腹腔注射苯巴比妥麻醉后，向膝关节腔内注射不同浓度的 MIA 溶液。<br><br>2 实验动物的处死及尸体的处理：<br>腹腔注射苯巴比妥麻醉后，腹主动脉取血处死，留取有关穴区皮肤及膝关节，无害化尸体处理。</td></tr>
<tr><td colspan="4">申请人签名：丁宁　　　　联系电话：15010237226</td></tr>
<tr><td colspan="4"></td></tr>
<tr><td>项目负责人承诺</td><td colspan="4">以上填写内容属实，本人对动物实验的设计的科学性、合理性和可行性负全责。<br>项目负责人签字：[illegible]　　　　日期：2017年5月15日</td></tr>
<tr><td>审查依据</td><td colspan="4">1. 该项目是否必须用实验动物进行实验，即能否用计算机模拟、细胞培养等非生命方法替代动物或用低等动物替代高等动物进行实验？<br>2. 表中所填申请人资格和所用动物的品种品系、质量等级、规格是否合适，能否通过改良设计方案或用高质量的动物来减少所用动物的数量？<br>3. 能否通过改进实验方法、调整实验观测指标、改良处死动物的方法，来优化实验方案、善待动物？</td></tr>
<tr><td rowspan="2">审查结果(是否同意申请人的实验方案)</td><td colspan="4">实施单位审核意见：<br>同意<br>签字：[illegible]　2017年5月17日</td></tr>
<tr><td colspan="4">北京中医药大学学术委员会实验动物伦理分委员会意见：<br>[stamp: 王之 印停]　（签章）　[stamp: 王之 印停]　2017年9月27日<br>[stamp: 北京中医药大学 医学与实验动物伦理委员会 110000003476]</td></tr>
</table>
